# Cleansing efficacy of an oral irrigator with microburst technology in adolescent orthodontic patients. A randomized-controlled crossover study

**DOI:** 10.1007/s00784-024-05842-9

**Published:** 2024-09-13

**Authors:** Hanna Gänzer, Manuel Kasslatter, Vera Wiesmüller, Lena Denk, Anna-Maria Sigwart, Adriano Crismani

**Affiliations:** 1 University Hospital of Orthodontics, Medical University of Innsbruck, Anichstr. 35, Innsbruck, 6020 Austria; 2 Department of Conservative Dentistry and Periodontology, Medical University of Innsbruck, Anichstr. 35, Innsbruck, 6020 Austria; 3 Südtirol Dental Clinic, Latsch, Italy

**Keywords:** Oral irrigator, Adolescent, Orthodontic, Oral hygiene, Plaque index, Gingival bleeding index, Fixed orthodontic treatment

## Abstract

**Objectives:**

Simplifying interdental space cleaning is a constantly discussed topic. The present study aimed to compare the cleansing efficacy of an oral irrigator with that of dental flossing in adolescent patients with fixed braces after four weeks of home-use.

**Materials and methods:**

The study design is a randomized, single-blinded cross-over study. Following a twenty-eight-day period of product utilization in a home setting, a comparative analysis was conducted on hygiene indices, the Rustogi Modified Navy Plaque Index (RMNPI) and the Gingival Bleeding Index (GBI), between the test group (oral irrigator) and the control group (dental floss).

**Results:**

Seventeen adolescent individuals completed the study. After 28 days of cleaning with the oral irrigator, RMNPI was 58.81% (55.31–66.47) compared to 59.46% (52.68–68.67) with dental floss (*p* = 0.070). Subgroup analyses did not indicate the superiority of either method. GBI after the test phase with the oral irrigator was 28.93% (23.21–33.97) and insignificantly higher compared to 26.40% (21.01–31.41) achieved with dental floss (*p* = 0.1585).

**Conclusions:**

Neither of the two products demonstrated statistically significant superiority in terms of cleaning efficacy. Therefore, no recommendation can be made in favor of one over the other. It was found that the high initial hygiene indices for fixed orthodontic appliances could be improved through increased awareness and precise instruction.

**Clinical relevance:**

For adolescent patients who struggle to use interdental brushes an oral irrigator may be suggested as a simple alternative in hard-to-reach areas, such as those around a fixed dental appliance.

## Introduction

The most important measure to prevent tooth decay is good oral hygiene. If the adherent bacterial film is regularly removed, further accumulation of germs and their sugar metabolism can be prevented [1]. Individuals who integrate interdental cleaning devices in their daily oral hygiene schedules experience fewer instances of dental caries, less periodontal diseases and have less missing teeth compared to those who exclusively employ electric or manual toothbrushes for oral hygiene [2]. Interdental brushes appear to offer superior effectiveness compared to dental floss [3]. As outlined in the consensus report compiled by the European Federation of Periodontology in 2015, the application of dental floss should be restricted to areas with gingival and periodontal health, where interdental brushes might pose a risk of causing traumatic injuries (2, [4]. The willingness to incorporate dental floss into the daily oral hygiene routine is quite limited. Additionally, the proper use of dental floss proves to be a significant challenge [5]. A noteworthy issue in the field of orthodontics is that in patients with fixed braces the accumulation of biofilm is promoted both above and below the gumline, compromising effective oral hygiene and consequently resulting in alterations in the oral microbiome, enamel decalcification, and the development of gingivitis [6–8]. Furthermore, several systematic reviews have consistently shown a decline of clinical parameters linked to periodontal diseases, including indicators such as the plaque index, bleeding on probing (BOP), attachment loss, and the development of pockets or gingival recessions [9, 10]. This deterioration has been associated with both the duration and the type of orthodontic treatment. [7, 11] Water flossers represent a recent innovation in interdental tools designed for regular use at home with the advantage of very easy application. Operating on the principles of pulsation and pressure, the water flosser disrupts plaque and removes loosely lodged debris. The primary use of water flossers is to assist individuals with reduced manual skills, but it may also be useful for patients undergoing orthodontic treatment. [12, 13] The available data regarding the use of oral irrigators in orthodontic patients is currently quite limited, and the results obtained from existing studies exhibit significant variations in terms of efficacy [14]. Most oral irrigators use water only, however there is the possibility to use a mixture of air and water, called microburst technology, to mechanically remove plaque. There has been no study specifically designed to evaluate the performance of an oral irrigator featuring microburst technology in adolescent orthodontic patients. The aim of this current randomized, single-blinded cross-over study was to assess and compare the cleaning effectiveness of microburst technology and dental flossing in adolescent individuals with fixed braces undergoing orthodontic treatment, following a 4-week period of at-home use. The null hypothesis postulates that there is no distinction between the two methods.

## Materials and methods

This study was approved by the Ethics committee of the Medical University of Innsbruck, Austria (ID AN 5123). The study was conducted in accordance with the 1964 Helsinki declaration and its later amendments. Prior to inclusion all subjects signed an informed written consent.

### Study subjects

Twenty minor subjects of the University Hospital of Orthodontic Dentistry, Innsbruck, Austria, were recruited in the period from October 2021 to March 2023. Inclusion criteria were fixed braces attached buccally at a minimum of four teeth per quadrant and existing contact points between all teeth. Exclusion criteria were pregnancy, oral or systemic diseases other than gingivitis, and the need for frequent drug consumption.

Due to time constraints, it was not possible to start with all 20 study participants simultaneously. As a result, the children were enrolled in the study in staggered groups. Data collection was performed from October 2021 to March 2023.

## Clinical intervention

The cleansing efficacy of the microburst technology (*Airfloss*^®^, Philips, Hamburg, Germany) versus interdental cleaning with dental floss (*Superfloss*^®^^,^ Oral-B, Boston, USA) was evaluated in a randomized-controlled, examiner-blinded, crossover study.

The study design consisted of four appointments for each subject at intervals of one month. During the first appointment, the probands received an explanation of the study procedure. The adolescents were assessed for study inclusion and exclusion criteria using the Case-Report-Forms (CRF) and an informed consent form was signed. Documentation of plaque-covered areas was evaluated using the Modified Navy Plaque Index developed by Rustogi (RMNPI) [15] subsequent to plaque disclosure (*2Tone*, Young, Earth City, Mo, USA) and the Gingival Bleeding Index after Ainamo & Bay (GBI) [16] was used for evaluation of the baseline hygiene indices.

The Rustogi Modified Navy Plaque Index (RMNPI) subdivides each buccal and lingual tooth surface into nine distinct segments (designated as A – I), which are subject to evaluation regarding the presence or absence of dental plaque. This particular index facilitates the differentiation of marginal regions of the dentition (A – C), interdental spaces (D, F), as well as the overall tooth surface areas (A – I). The RMNPI is computed as the proportion of biofilm-adhering sites relative to the total number of assessed sites. In the context of the assessment of the Gingival Bleeding Index (GBI), a periodontal probe (PCP 12, Hu Friedy, Chicago, USA) was inserted into the gingival sulcus. This instrument is utilized to dichotomously determine, at six distinct sites per tooth (mesiobuccal – buccal – distobuccal – mesiolingual – lingual – distolingual), whether bleeding is elicited or not. The percentage of bleeding sites in relation to the total number of measured sites was calculated. It is noteworthy that teeth that weren’t integrated into the fixed orthodontic treatment were excluded from the analysis. Moreover, all examinations were conducted by a single trained examiner.

Prior to commencing the investigation, randomization of the test products was carried out using a computer-generated method within *Microsoft*^®^ Office Excel. The randomization process was carried out by study assistants, who also provided thorough hands-on training to the study participants to ensure that the examiner was blinded. Following the manufacturer’s recommended protocol for the oral irrigator with microburst technology (*Airfloss*^®^, Philips, Hamburg, Germany), the device was filled with water and activated once per interdental space, utilizing the default setting of three sprays per activation. For the control products (*Superfloss*^®^, Oral-B, Boston, USA) participants were instructed to thread it from the buccal side below the orthodontic wire and position it around the tooth in a c-shaped manner to facilitate cleaning in the apico-coronal direction. In relation to toothbrushing, participants were directed to maintain their customary oral hygiene routine and use their preferred products. After comprehensive instruction regarding the initial randomly assigned test product, professional tooth cleaning was carried out using an air-polishing device (*Airflow*^®^ prophylaxis master and *Airflow*^®^ Plus powder; both EMS, Nyon, CH), supplemented by using sonic scalers and rubber cups with polishing paste (*Cleanic*^®^, Kerr, Bioggo, CH) as needed.

After a test period of twenty-eight days employing the first test product, study participants attended their second visit to reassess hygiene indices and inclusion/exclusion criteria. Subsequently, there was a washout phase lasting twenty-eight days during which the subjects reverted to their usual oral hygiene procedures. Following this period, they returned for the third visit. Once again, plaque disclosure was performed, and subjects were given detailed instructions for the utilization of the second product, followed by another professional dental cleaning. In a manner consistent with the first test phase, participants utilized the second product for twenty-eight days, then presented for an examination of the plaque- and gingival index during the fourth and final appointment of the study.

### Statistical analysis

The sample size calculation relied on the average values and standard deviations of overall plaque scores analyzed in a study conducted by Heiß-Kisielewsky et al., which aimed to compare the efficacy of microburst technology (Airfloss^®^, Philips, Hamburg, Germany) with dental flossing. [17] The sample size calculation for dependent samples, with a power of 80% and α = 0.05, resulted in a sample size of 16. Accounting for an assumed drop-out rate of 25%, the final sample size was *n* = 20.

At the individual level, RMNPI values were determined by dividing the total number of areas with plaque present by the total number of assessed sites. These values were then compared between the two tooth-cleaning techniques using the Wilcoxon signed-rank test. The gingival bleeding index was computed in a similar way. Unless indicated otherwise, median and interquartile range were provided. The significance level was established at *p* < 0.05.

## Results

Twenty individuals were recruited. Seventeen participants (seven females and ten males) finished the study with a mean age of 14.76 ± 1.15 (minimum 14 to maximum 18) years. The drop-out rate was 15%. Three participants could not continue due to systemic antibiotic therapy, illness, and non-compliance, and were therefore excluded from the study.

### Plaque scores

**Fig. 1:**
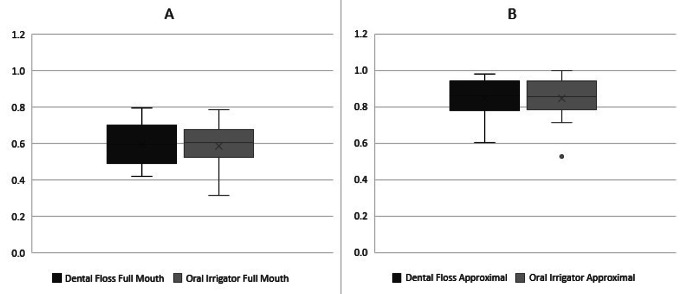
Rustogi Modified Navy Plaque Index (RMNPI) after 28-days of home-use of dental floss in comparison to an oral irrigator. Rustogi modified plaque-index splits every buccal and lingual tooth surface into nine sections (A – I) and was calculated as percentage of biofilm adhering sites to measured sites of **(A)** all tooth surfaces (A – I), and **(B)** approximal surfaces (D, F)

After 28 days of interdental cleaning with microburst technology, the median of overall RMNPI was reduced from baseline 69.56% (56.06–76.59) to 60.71% (55.31–66.47) (*p* = 0.037). Dental flossing for 28 days after a median baseline value of 71.79% (69.44–78.53) resulted in a RMNPI of 59.72% (52.68–68.67). There was no statistically significant difference found between the plaque scores of the test products (*p* = 0.250) and the initial values (*p* = 0.070) (see Fig. 1).

Subgroup analysis, differentiating between approximal surfaces, marginal surfaces, anterior, or posterior teeth, did not reveal any statistically significant differences in plaque scores (*p* > 0.05) (see Table 1). As shown in Fig. 1 there was no difference in plaque index after 28 days of dental flossing compared to microburst technology on approximal areas (median 86.36% and 85.71%, respectively; *p* = 0.704). (see Fig. 1)

R*MNPI*, Rustogi Modified Navy Plaque Index; *%*, percent; *, *p* value < 0.05.

### Gingival bleeding index

**Fig. 2:**
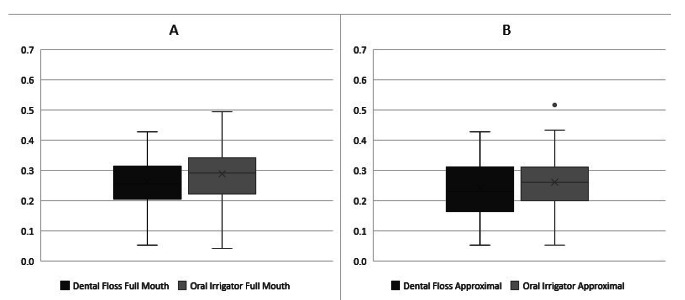
Gingival Bleeding Index after 28-days of home-use of a dental floss in comparison to an oral irrigator. Gingival bleeding Index was calculated dichotomously as percentage of bleeding sites to measured sites of **(A)** all tooth surfaces, **(B)** approximal surfaces

The median full mouth GBI after 28 days of interdental cleaning with the oral irrigator did not show a statistically significant change from baseline, with values of 29.33% (25.00–40.74) and 29.17% (23.21–33.97) respectively (*p* = 0.509). Dental flossing for 28 days after a median baseline value of 35.26% (28.85–42.49) resulted in a GBI of 25.60% (21.01–31.41) (*p* = 0.001). The bleeding scores of the test products did not show a statistically significant difference (*p* = 0.230). Similarly, there was no statistically significant difference found between the bleeding scores of the initial bleeding scores before intervention (*p* = 0.158) (see Fig. 2). Subgroup analysis revealed that the gingival bleeding index was not statistically significantly different on approximal sites after 28 days of interdental cleaning with floss compared to microburst technology (median 23.21% and 26.14%, respectively; *p* = 0.529) (see Fig. 1).

There were no statistically significant differences in GBI when analyzing only the proximal surfaces, anterior or posterior teeth (*p* > 0.05) (see Table 1).

**Table 1:** Plaque and bleeding levels after one month of home use. The Rustogi modified plaque-index splits every buccal and lingual tooth surface into nine sections (A – I) and was calculated as percentage of biofilm adhering sites to measured sites. Gingival bleeding was calculated dichotomously at 6 sites per tooth as percentage of bleeding sites to measured sites. Data was presented using median and interquartile ranges

	Microburst technology	Dental flossing	*p*-value
**Full mouth**			
RMNPI (%)	60.71% (55.31–66.47)	59.72% (52.68–68.67)	0.070
Gingival bleeding index (%)	29.17% (23.21–33.97)	25.60% (21.01–31.41)	0.158
**Approximal sites**			
RMNPI (%)	85.71% (83.93–94.32)	86.36% (78.85–93.75)	0.704
Gingival bleeding index (%)	26.14% (20.37–31.00)	23.21% (17.86–30.56)	0.529
**Approximal buccal sites**			
RMNPI (%)	94.64% (87.50–97.83)	94.64% (85.19–97.92)	0.905
Gingival bleeding index (%)	18.18% (9.62–28.00)	19.64% (13.64–30.36)	0.912
**Approximal lingual / palatal sites**			
RMNPI (%)	82.69% (71.43–94.64)	82.14% (75.00–91.07)	0.624
Gingival bleeding index (%)	27.27% (22.22–44.23)	25.00% (19.64–32.69)	0.271
**Anterior Teeth**			
RMNPI (%)	62.04% (54.63–70.37)	60.32% (49.54–73.15)	0.596
Gingival bleeding index (%)	26.39% (24.24–33.33)	22.92% (20.83–30.56)	0.453
**Posterior Teeth**			
RMNPI (%)	57.64% (48.99–64.93)	57.29% (50.17–63.10)	0.337
Gingival bleeding index (%)	29.73% (23.21–33.97)	25.44% (19.23–32.29)	0.168

RMNPI, Rustogi Modified Navy Plaque Index; %, percent

## Discussion

It is the daily challenge of interdental tooth cleaning for patients undergoing orthodontic treatment, which must be combated using simplified cleaning methods.

In addition to dental floss, other oral hygiene aids such as interdental brushes or water flossers are available. However, especially in young people, the spaces between the teeth are often too narrow for the use of interdental brushes and the orthodontic wires makes it difficult to use dental floss. The result is gingivitis and white spot lesions as a common side effect of fixed orthodontic treatment. [7, 8, 15]

The existing research regarding the efficacy of dental floss and water flossers is quite varied. Several industry-funded studies have suggested that water flossers provide better cleaning results and more reduction of inflammation than flossing or interdental brushes [18, 19], while Worthington et al. expressed uncertainty about the effectiveness of oral irrigators in reducing gingival inflammation [3]. Data on oral irrigators in orthodontic patients is limited. On one hand, the superiority of an oral irrigator with an orthodontc tip over dental floss could be demonstrated in reducing gingival bleeding (*p* < 0,001) [20]. On the other hand a previous similarly designed trial, which concentrated on adult patients with fixed orthodontic appliances, found that dental floss provided more efficient cleaning results, especially with regard to reducing gingival bleeding [21]. However, in this current study no statistically significant differences were observed between microburst technology and dental flossing neither in terms of plaque reduction (60.71% (55.31–66.47), respectively 59.72% (52.68–68.67); *p* = 0.070) nor in regard of gingival bleeding (29.17% (23.21–33.97), respectively 25.60% (21.01–31.41); *p* = 0.158).

In this study we used the Rustogi Modified Navy Plaque Index [22] for evaluating plaque presence or absence in nine areas on buccal or lingual tooth surfaces. The authors decided to use a dichotomous index due to better analyzability from a statistical point of view. The RMNPI allows to measure plaque levels on a full-mouth level, but also subgroup analyses including smooth surfaces, interdental and gingival margin areas. Based on the lack of statistically significant difference found in any subgroup (*p* > 0.05) (see Table 1), the use of an oral irrigator in adolescents with narrow interdental spaces can be recommended because of its easy-to-use application.

A limiting factor of the significance of this study undoubtedly resides in the small number of participants. The selection process posed challenges due to the inclusion criteria specifying an age of over 14 years, which naturally, constrained the pool of eligible patients. Many younger patients receiving treatment at the University Clinic of Orthodontics were thereby excluded from the study. Additionally, the age of the participants played a role in their decreased motivation and interest in maintaining oral hygiene. Furthermore, many adolescents do not receive proper instructions or monitoring from their parents regarding oral hygiene. This made it difficult to motivate the study participants to accurately use the products.

Another study design´s limitations may include the potential decrease in gingival bleeding index due to professional cleaning, which could diminish the comparability with the baseline gingival bleeding index. In order to achieve a consistent baseline value before the first and second test phase, a washout period of 28 days was chosen, during which participant maintained their original oral hygiene routine.

In conclusion and as mentioned before, none of the two products proved significantly superior in terms of cleaning efficacy and reduction of gingival bleeding. Neither of the two products demonstrated statistically significant superiority in terms of cleaning efficacy. Therefore, no recommendation can be made in favor of one over the other. It was found that the high initial hygiene indices for fixed orthodontic appliances could be improved through increased awareness and precise instruction. For adolescent patients who struggle to use interdental brushes an oral irrigator may be suggested as a simple alternative in hard-to-reach areas, such as those around a fixed dental appliance.

## Data Availability

No datasets were generated or analysed during the current study.
